# Evaluation of multidrug resistance patterns in siderophore-producing *Pseudomonas aeruginosa* from clinical and environmental samples in Gorgan, Iran

**DOI:** 10.1016/j.nmni.2018.04.003

**Published:** 2018-04-21

**Authors:** M. Sadeqi Nezhad, H. Pordeli, N. Ghasemi, A. Ahani

**Affiliations:** 1) Young Researchers and Elites Club, Gorgan Branch, Islamic Azad University, Gorgan, Iran; 2) Department of Laboratory Science, Gorgan Branch, Islamic Azad University, Gorgan, Iran; 3) Department of Microbiology, Gorgan Branch, Islamic Azad University, Gorgan, Iran

**Keywords:** Antibiotic resistance, clinical, *Pseudomonas aeruginosa*, siderophore

## Abstract

Siderophores secreted by nonfermentative negative bacilli such as *Pseudomonas aeruginosa* are capable of increasing rates of resistance to carbapenem antibiotics. Furthermore, the resistance of these isolates to antibiotics has been enhanced by producing siderophores, and their frequencies have erratic patterns. We studied the outbreak of *P. aeruginosa* strains and their antibiotic patterns in different clinical samples. In this descriptive cross-sectional study, 100 *P. aeruginosa* samples were isolated from different clinical specimens at the 5th Azar Hospital, Gorgan, Iran, in 2017. These strains were identified by biochemical tests, and their antibiotic resistance patterns were measured via the disc diffusion method. Next imipenem and EDTA-imipenem (10–30 μg) antibiotics were employed for the detection of siderophores. Amongst 100 *P. aeruginosa* samples, 31 isolates (31%) were siderophore carriers. The frequency of this enzyme among specimens was as follows: 56.2% in burn wounds, 36.4% in urine, 22.2% in respiratory secretion, 19.4% in blood and 16.7% in wounds (p > 0.05). Moreover, *P. aeruginosa* isolates producing siderophores had the highest range of resistance to ciprofloxacin (47.6%), gentamicin (46.7%), ceftazidime (34.9%), nalidixic acid (34.3%), amikacin (34.1%) and cefotaxime (31.6%). The prevalence of siderophore producers, and especially their antibiotic patterns have no specific algorithms; in addition, an antibiogram is recommended to identify the most effective antibiotics against those isolates.

## Introduction

Iron is a principal substance required by microorganisms to develop. Several mechanisms exist to acquire iron for hosts; one common way to gain iron is by siderophore secretion. Siderophores are small molecules secreted by *Pseudomonas aeruginosa*, a negative bacilli bacteria. They play an important role in microorganisms and are essential for the process and development of DNA synthesis and iron chelating because of their capability to transfer iron into the bacterium. *P. aeruginosa* can be found or grown in soil, water, antiseptic detergents, and animal and plant tissues; they are stable in difficult growing situations and in hospitals [1].

At present, multidrug resistance is developing. Opportunistic pathogens such as *P. aeruginosa* may cause infections in humans with immunodeficiency, particularly in the setting of hospitals. These bacteria are the most common bacilli causing adverse effects isolated from infections in blood, trauma sites and pneumonia, as well as in the genitourinary system and intra-abdominally [2], [3]. Moreover, *P. aeruginosa* is the second most common cause of pathogenic bacteria in surgery and the third most common hospital infection, after *Escherichia coli* and *Staphylococcus aureus* respectively, with almost 10% of hospital infections attributed to it [4], [5]. *P. aeruginosa* infection is severe; it causes death in patients with bacteraemia and pneumonia at rates of 30–50% and 45–70% respectively [6].

*P. aeruginosa* needs three uptake systems such as pyochelin, pyoverdine and ferripyochelin when confronted with iron deficiency in hosts. Pyoverdine synthesis plays an essential role in growth in human serum, whereas the two compounds stimulate growth in iron-deficient conditions. The increase in of multidrug resistance is a significant cause of abnormality in majority of patients that make difficulty in treatment and also augments the mortality and morbidity [7]. The diverse capability of *P. aeruginosa* in infection indicates that this strain may possess a specific genome. Its chromosomes are enriched with G+C (50–70%); genome sequencing conducted on 2000 *P. aeruginosa* samples demonstrated that the genome comprises 6.3 million base pairs and 5570 genes, although 8.4% of these genes are regulatory genes that cause compatibility and illness in these bacteria [8].

We assessed the frequency of *P. aeruginosa* in various clinical samples and analysed their antibiotic patterns.

## Materials and methods

This study assessed 100 *P. aeruginosa* samples from various clinical specimens, including blood, respiratory secretions, urine, wound, pus swab and burn wounds; and from various environments, including intensive care units, burn units, surgery units, internal medicine units and paediatric neurology. All samples were collected from the 5th Azar Hospital in Gorgan, Iran, during 2017.

All subjects provided written informed consent. Demographic data were collected, including age, gender, unit, type of specimen and mortality. Then isolates were transported to the microbiology laboratory under sterile conditions. They were cultured onto MacConkey medium and incubated at 37°C for 18 to 24 hours. Grown colonies were identified as *P. aeruginosa* via biochemical means, including positive oxidase test, negative Gram bacilli, grown in cetrimide agar, grown at 42°C and non–lactose fermenting.

One colony of *P. aeruginosa* was taken and cultured onto Mueller-Hinton broth, then incubated at 35°C for one night. A sub McFarland suspension of the colony was prepared and cultivated on Mueller-Hinton agar. Disc diffusion was performed, with the incubation method including processing at 37°C; then after 18 to 24 hours the sensitivity and resistance of isolates to antibiotics was observed and calculated according to Clinical and Laboratory Standards Institute (CLSI) guidelines. Tested antibiotics included gentamicin, ceftazidime, cotrimoxazole, ciprofloxacin, ceftriaxone, nalidixic acid, amikacin, cefotaxime and imipenem. All antibiotics were purchased from the Mast Group (Bootle, UK), and *P. aeruginosa* ATCC22853 was used as the control strain.

For the detection of siderophore carrier strains, imipenem and EDTA-imipenem (10–30 μg) antibiotics were used. At this stage the isolates were selected because of their resistance to imipenem. The strains were cultured on Mueller-Hinton agar with imipenem and EDTA-imipenem (10–30 μg) to a distance of 25 mm. After 18 to 24 hours' incubation at 37°C, according to the CLSI protocol, the siderophore carrier strains were identified according to their sensitivity to EDTA-imipenem (10–30 μg) being ≥7 mm compared to imipenem alone.

## Results

Among 100 *P. aeruginosa* strains, 31 (31%) were identified as siderophore carriers. We ascertained the frequency of siderophores in various specimens and units. We found that of the 31 total siderophore-producing isolates, 12 were from urine, four were from respiratory secretions, two were from wounds, nine were from burn wounds and four were from blood (Table 1). There was no significant correlation between specimens and the presence of siderophores (p 0.129). The incidence of organisms producing siderophores in different units found the highest rate in the internal medicine (45.8%) and burn (45.8%) units; the lowest rate was observed in the paediatric neurology (9.2%) and surgery (15.8) units (p 0.058).

**Table 1:** Frequency of siderophore-producing isolates among various specimens

Sample	Total, *n* (%)	No siderophores, *n* (%)	Siderophores, *n* (%)
Urine	33 (100)	21 (63.6)	12 (36.4)
Respiratory secretion	18 (100)	14 (77.8)	4 (22.2)
Wound	12 (100)	10 (83.3)	2 (16.7)
Burn wound	16 (100)	7 (43.8)	9 (56.2)
Blood	21 (100)	17 (80.96)	4 (19.4)
Total	100 (100)	69 (69)	31 (31)

**Fig. 1:**
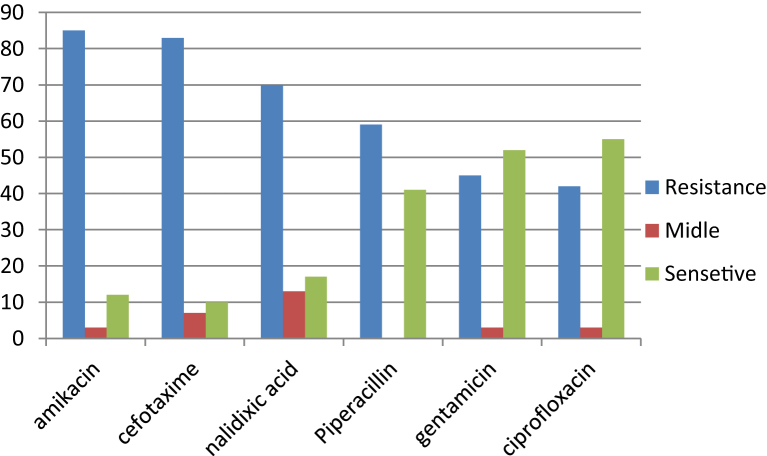
Antibiotic resistance and sensitivity patterns among isolates.

Fig. 1 shows the frequency of antibiotic resistance among 100 isolates; the most resistance was to amikacin, ceftazidime and cefotaxime. The highest sensitivities were observed in ciprofloxacin, gentamicin and nalidixic acid. Table 2 lists the resistance and sensitivity patterns of siderophore-producing isolates.

**Table 2:** Frequency of resistance patterns in various antibiotics among siderophore-carrying isolates

Antibiotic	Siderophores, *n* (%)	No siderophores, *n* (%)
Gentamicin	21 (46.7)	24 (53.3)
Amikacin	29 (34.1)	56 (65.9)
Ciprofloxacin	20 (47.6)	22 (52.4)
Nalidixic acid	24 (34.3)	46 (65.7)
Cefotaxime	25 (31.6)	54 (68.4)
Ceftazidime	29 (34.9)	54 (65.1)

## Discussion

The introduction of carbapenems to the field of medicine was a significant moment in the treatment of serious bacterial infections caused by siderophore-carrying bacteria because of their extensive range of activity and stability against most siderophores [9]. Resistance to carbapenems has been observed in most nonfermenting strains such as *P. aeruginosa*. The most frequent resistance mechanisms are penetration reduction and the production of hydrolyzing siderophores.

In this study 31 siderophore carriers were isolated from 100 specimens. According to two different studies in Iran 11.1% and 8% of isolated *P. aeruginosa* were siderophore carriers [10], [11]. Although in the present study the frequency of siderophore carriers was higher, it indicates that in our province, a variety of antibiotics have been used which has influenced the incidence of siderophores. Two studies in France and Spain demonstrated that 46% of isolates were siderophore carriers—a higher frequency than in this study [12], [13]. The *P. aeruginosa* specimens isolated from a burn unit in Kurdistan indicated that 22% of burn-wound samples were siderophore carriers [14]. However, the rate at which our study isolated *P. aeruginosa*–producing siderophores from burn-wound culture was much higher, at 56.4%. This may show that using imipenem and vancomycin for prophylaxis in burn wounds at different burn units could affect the incidence of siderophores in hospitals. Further, in a study that sought to detect and classify siderophore-producing isolates, 70 *P. aeruginosa* samples isolated from an intensive care unit found that 78% were siderophore producers [15]. The incidence of siderophore producers was lower in our study than theirs. Norozi et al. [11] reported that 8% of isolates in a burn unit were positive for siderophores. However, in our study, the rate was higher. Among siderophore-positive isolates from various clinical samples from hospital patients in our study, maximum resistance was observed for ciprofloxacin, followed by gentamicin, ceftazidime, nalidixic acid, amikacin and cefotaxime. For siderophore-negative isolates, maximum resistance was observed for cefotaxime, followed by amikacin, nalidixic acid, ceftazidime, gentamicin and ciprofloxacin. This is in contrast to the study of Kalantar et al. [16], for which ceftazidime (96%), nalidixic acid (89%) and cefotaxime (88%) had the most resistance.

## Conclusion

Performing an antibiogram before prescribing antibiotics is crucial to reducing multidrug resistance. Further, confronting multidrug-resistant isolates in burn units and intensive care units must be done because they have the greatest adverse effect on health.

## Conflict of interest

None declared.
